# Apoptotic Caspases Suppress Expression of Endogenous Retroviruses in HPV31+ Cells That Are Associated with Activation of an Innate Immune Response

**DOI:** 10.3390/v16111695

**Published:** 2024-10-30

**Authors:** Caleb Studstill, Ning Huang, Shelby Sundstrom, Samantha Moscoso, Huirong Zhang, Blossom Damania, Cary Moody

**Affiliations:** 1 Department of Microbiology and Immunology, University of North Carolina at Chapel Hill, Chapel Hill, NC 27599, USA; 2 Lineberger Comprehensive Cancer Center, University of North Carolina at Chapel Hill, Chapel Hill, NC 27599, USA

**Keywords:** human papillomavirus, viral life cycle, innate immunity, endogenous retrovirus

## Abstract

Avoidance of an immune response is critical to completion of the human papillomavirus (HPV) life cycle, which occurs in the stratified epithelium and is linked to epithelial differentiation. We previously demonstrated that high-risk HPVs use apoptotic caspases to suppress an antiviral innate immune response during the productive phase of the life cycle. We found that caspase-8 and caspase-3 suppress a type I IFN-β and type III IFN-λ response by disabling the MDA5/MAVS double-stranded RNA (dsRNA) sensing pathway, indicating that immunogenic RNAs increase upon differentiation in HPV+ cells. In this study, we demonstrate that caspase inhibition results in aberrant transcription of a subset of endogenous retroviruses (ERVs) that have been shown to activate an IFN response through dsRNA-sensing pathways. We show that the increase in ERV transcription is accompanied by an enrichment in dsRNA formation. Additionally, we demonstrate that the robust increase in ERV expression requires activation of the JAK/STAT-signaling pathway, indicating that this subset of ERVs is IFN-inducible. Overall, these results suggest a model by which caspase activity blocks the reactivation of ERVs through the JAK/STAT pathway, protecting HPV+ cells from an increase in immunogenic dsRNAs that otherwise would trigger an IFN response that inhibits productive viral replication.

## 1. Introduction

Human papillomaviruses are small, double-stranded DNA viruses that exhibit a strict tropism for cutaneous or mucosal epithelial cells. Mucosal HPVs are divided into high-risk and low-risk categories based on their association with cancer. About 12–15 HPVs are considered high-risk and are the causal agents of cervical cancer, with 99% of cervical cancers containing high-risk HPV DNA. High-risk HPVs are associated with other genital malignancies as well as an increasing number of head and neck cancers, which are predominantly HPV16+ [1].

The life cycle of HPV occurs in the stratified epithelium and is characterized by three phases of replication regulated by the state of epithelial differentiation [2,3]. HPVs gain access to the basal, proliferating layer of the epithelium through a microwound and establish infection at a low copy number through a rapid amplification process. In these undifferentiated, proliferating cells, the virus is maintained at around 50–100 episomal copies per cell [4]. Epithelial differentiation triggers the productive phase of the life cycle, which consists of viral genome amplification to 100 s–1000 s of copies per cell, along with late viral gene expression, including expression of the L1 and L2 capsid genes, leading to virion assembly and release from the uppermost layers of the stratified epithelium [5,6,7]. The process of epithelial differentiation results in an exit from the cell cycle, and the cells cease to proliferate as they form the cornified outer layer. However, the limited coding capacity of the HPV genome renders the virus reliant on cellular factors for viral replication [2]. To establish an environment conducive to viral replication, HPV, largely through the actions of the E7 protein, pushes differentiating cells back into the cell cycle [8,9]. Productive replication subsequently occurs post-cellular DNA synthesis in a G2-arrested environment, allowing HPV access to cellular resources for replication without competition from the host [10]. How the cellular environment is further modified by HPV upon differentiation to support viral replication is still largely undefined.

One mechanism by which HPV establishes a replication-competent environment in differentiating cells is through the sub-lethal activation of apoptotic caspases [11,12]. Caspases are cysteine proteases that become activated through two converging apoptotic pathways: extrinsic (death-receptor mediated) and intrinsic (mitochondrial-mediated) [13]. The extrinsic pathway is initiated by the binding of death receptor ligands to their cognate death receptors, resulting in the activation of the initiator caspase, caspase-8 [14]. The intrinsic pathway is initiated by various cell stresses and results in mitochondrial outer membrane permeabilization (MOMP) by the pro-apoptotic molecules Bax and Bak [15]. MOMP results in the activation of the initiator caspase, caspase-9. Caspase-8 and caspase-9 both cleave and activate the effector caspase-3 and caspase-7, which cleave various cellular substrates that culminate in cell death [16]. Previous studies showed that high-risk HPV31 induces sub-lethal activation of caspase-8 as well as caspase-9, caspase-3, and caspase-7 upon differentiation, which is not observed in uninfected cells [11,12]. Treatment of differentiating HPV31+ cells with a pancaspase inhibitor or specific inhibitors of caspase-8, caspase-9, or caspase-3 blocks productive viral replication, identifying a pro-viral role for apoptotic caspases in the HPV life cycle [11,12].

We recently demonstrated that apoptotic caspase activity facilitates productive viral replication by suppressing an antiviral innate immune response [12]. Innate immune responses are initiated by pattern recognition receptors (PRRs) that sense pathogen-associated molecular patterns (PAMPs) as well as damage-associated molecular patterns (DAMPs), most commonly nucleic acids [17]. Activation of PRR–adaptor pairs leads to the production of interferon (IFN) and IFN-stimulated genes (ISGs) that establish an antiviral environment. Some of the most widely studied PRR pairs are the RNA-sensing RIG-I-like receptors, MDA5 and RIG-I, with their adaptor MAVS, and DNA-sensing cGAS with its adaptor STING [18,19]. Activation of these pathways leads to an IFN response through the phosphorylation and activation of the transcription factor IRF3 by the kinase TBK1. Apoptotic caspases maintain apoptosis as an immunologically silent form of cell death by suppressing a type I IFN response [20,21,22]. Caspases achieve this by cleaving components of DNA-sensing pathways (e.g., cGAS, IRF3) as well as RNA-sensing pathways (e.g., MAVS, IRF3), in turn suppressing IFN production and signaling [23,24].

Our previous studies demonstrated a critical role for caspase-8 and caspase-3 in suppressing a type I IFN-β and type III IFN-λ response in differentiating HPV31+ and HPV16+ cells [12]. While HPV induces a modest increase in IFN expression upon differentiation, we showed this response is exacerbated upon caspase inhibition. The increase in IFN expression is accompanied by a significant increase in the production and secretion of IFN-β and IFN-λ that act in a paracrine manner to induce the expression of ISGs, resulting in a block in productive viral replication. Interestingly, we found that the IFN response occurs in a manner dependent on the MDA5/MAVS dsRNA-sensing pathway rather than the cGAS/STING DNA-sensing pathway. These studies indicate that HPV hijacks a non-cell death function of apoptotic caspases to suppress an IFN response to immunogenic dsRNA. However, the source of the dsRNA that stimulates this response was not identified.

Several studies have shown that the activation of human endogenous retroviruses (ERVs) play an important role in stimulating an innate immune response [25]. ERVs arose from ancient retroviral infections and are widely distributed across our chromosomes, constituting approximately 8% of the human genome [26]. ERVs contain long terminal repeats (LTRs), which drive ERV expression, but are typically silenced by heterochromatin maintenance factors such as DNA methyltransferases (DNMT) and histone methyltransferases [25,27]. However, ERVs can be reactivated under certain cellular stresses, including exogenous viral infections [28]. Recent studies have shown that DNA hypomethylation induced by treatment with the DNMT inhibitor 5-aza-2-deoxytidine reactivates ERV expression, resulting in the formation of dsRNA that triggers a type I and type III IFN response through the MDA5/MAVS RNA-sensing pathway [29,30].

HPV is well established to globally alter the transcriptional profile of infected cells through epigenetic perturbations, including DNA methylation and histone post-translational modifications [31,32]. Episomal HPV DNA becomes hypomethylated upon differentiation [33], suggesting that DNMT activity may be altered. We therefore reasoned that epithelial differentiation may reactivate ERV expression to stimulate an MDA5-mediated antiviral response that is dismantled by caspase activity. In this study, we explored the interplay between caspase activity and the innate immune response to determine if ERV transactivation could play a role in eliciting an IFN response during the productive phase of the HPV life cycle.

## 2. Materials and Methods

### 2.1. Cell Culture and Reagents

Human foreskin keratinocytes (HFKs) were isolated from neonatal foreskins as previously described and maintained in DermaLife keratinocyte growth medium (KGM; Lifeline Cell Technology, Frederick, MD, USA), as previously described [34]. CIN612 9E cells were cultured in E medium supplemented with 5 ng/mL mouse epidermal growth factor (EGF, BD Biosciences, Franklin Lakes, NJ, USA) in the presence of mitomycin C-treated J2 3T3 fibroblast feeder cells, as previously described [35]. J2 fibroblast feeder cells were removed from HPV-positive cells by incubating in Versene (phosphate-buffered saline (PBS) supplemented with 1 mM EDTA) as necessary. J2 fibroblasts were cultured in DMEM with 10% bovine growth serum. The pancaspase inhibitor IDN-6556 (#HY-10396), caspase-8 inhibitor (Z-IETD-FMK) (#HY-101297), and caspase-3 inhibitor (Z-DEVD-FMK) (#HY-12466) were purchased from MedChemExpress (Monmouth Junction, NJ, USA), and ruxolitinib was purchased from Selleckchem (Houston, TX) (#INCB018424).

### 2.2. Calcium-Induced Differentiation

For calcium-induced differentiation, sub-confluent HFKs and CIN612 9E cells were cultured in serum-free keratinocyte basal medium (KBM, Lonza (Walkersville, MD, USA)) containing growth supplements overnight and then changed to KBM media (without supplements) containing 1.5 mM calcium chloride, as previously described [11]. Cells were cultured in high-calcium medium for the indicated time points. RNA and protein were harvested at indicated timepoints for further analysis.

### 2.3. Western Blotting

Cells were lysed in RIPA buffer (50 mM Tris, pH 7.5, 150 mM NaCl, 1 mM EDTA, 1% Nonidet P-40, 0.1% SDS) supplemented with Complete Mini protease inhibitor (Roche, Indianapolis, IN (Fre) and PhoSTOP phosphatase inhibitor tablets (Roche). Equal amounts of protein were electrophoresed using SDS-polyacrylamide gels and subsequently transferred to polyvinylidene difluoride (PVDF) membranes (Millipore, Burlington, MA, USA). Membranes were probed with the following primary antibodies: Involucrin (Santa Cruz, Dallas, TX, #sc-398952, 1:1000), DNMT1 (Cell Signaling Technology, Danvers, MA, #5032, 1:1000), total STAT1 (Cell Signaling #14994, 1:1000), pSTAT1 Tyr701 (Cell Signaling #9167, 1:1000), total STAT2 (Cell Signaling, #7260, 1:1000), pSTAT2 Tyr690 (Cell Signaling, #88410, 1:1000), and GAPDH (Santa Cruz, #sc-365062, 1:4000). Membranes were then incubated with Horseradish peroxidase (HRP)-conjugated secondary antibodies (GE Life Sciences, Marlborough, MA, USA) for one hour at room temperature. Western blots were developed using Clarity Western ECL blotting substrate (Bio-Rad, Hercules, CA, USA). Images were captured with either autoradiography film or the Biorad ChemidocMP imaging system and analyzed with Biorad ImageLab 6.1 software.

### 2.4. Real-Time PCR

Total RNA was extracted using RNA Stat 60 (Tel-Test Inc., Friendswood, TX, USA). A total of 1 ug RNA was treated with DNase I (Invitrogen, Waltham, MA, USA) followed by cDNA synthesis using the Superscript VILO reverse transcription kit (Invitrogen) per the manufacturer’s instructions. qPCR was performed in triplicate using 50 ng of cDNA and the Applied Biosystems QuantStudio 6 Flex real-time PCR thermal cycler (ThermoFisher, Waltham, MA, USA) and SsoAdvanced Universal SYBR Supermix (Bio-Rad). The thermal profile used for PCR is as follows: 10 min denaturation at 95 °C followed by 40 cycles of 95 °C for 15 sec, 60 °C for 30 sec. Melt curves were subsequently performed to ensure proper primer annealing. Relative transcript levels were determined using the threshold cycle method (2^−ΔΔCT^) with GAPDH as an endogenous control gene. The primer sequences used are as follows:

MLTA10: Forward TCTCACAATCCTGGAGGCTG;
Reverse GACCAAGAAGCAAGCCCTCA
MLT1B: Forward TGCCTGTCTCCAAACACAGT;
Reverse TACGGGCTGAGCTTGAGTTG
MER21C: Forward GGAGCTTCCTGATTGGCAGA;
Reverse ATGTAGGGTGGCAAGCACTG
MER4D: Forward CCCTAAAGAGGCAGGACACC;
Reverse TCAAGCAATCGTCAACCAGA
IFN-β: Forward CAGCAATTTTCAGTGTCAGAAGC;
Reverse TCATCCTGTCCTTGAGGCAGT
GAPDH. Forward, 5′-CTGTTGCTGTAGCCAAATTCGT-3′;
Reverse, 5′-ACCCACTCCTCCACCTTTGAC-3′

### 2.5. RNA Immunoprecipitation (IP)

CIN612 cells were differentiated for 72 h in high-calcium medium in the presence of DMSO or 10 μM IDN-6556 (pancaspase inhibitor). Total RNA was isolated using TRIzol (ThermoFisher) and RNA precipitation was carried out as described previously [36]. Briefly, 50 µg of total RNA was diluted in 100 µL of NET2 Buffer (50 mM Tris–HCl pH 7.6, 150 mM NaCl, 3 mM MgCl2) and treated with 2 units of RNase I (Thermo AM229) and 2 µL of TURBO DNase (Thermo AM2238) at 37 °C for 10 min. Next, 5 µg of J2 anti-dsRNA antibody (SigmaAldrich, St. Louis, MO, USA) and 200 units of RNase inhibitor (ThermoFisher AM2696) were added to the immunoprecipitation (IP) fraction and incubated overnight at 4 °C, as previously described [37]. Protein G Dynabeads (Thermo 10004D) were added to the J2 immunoprecipitated mixture and incubated for two hours. The beads were washed four times with NET2 buffer, and the bound RNA was isolated using TRIzol LS (Thermo) and purified with the RNA Clean and concentrator (Zymo Research, Irvine, CA). The eluted RNA was incubated at 70 °C for 3 min and then reverse transcribed using the SensiFAST cDNA Synthesis Kit (Bioline, Memphis, TN, USA). Real-time qPCR was performed, and enrichments were calculated using 1% of the input RNA as a reference for each immunoprecipitation.

## 3. Results

### 3.1. Expression of Endogenous Retroviruses (ERVs) Increases upon Differentiation

**Figure 1:**
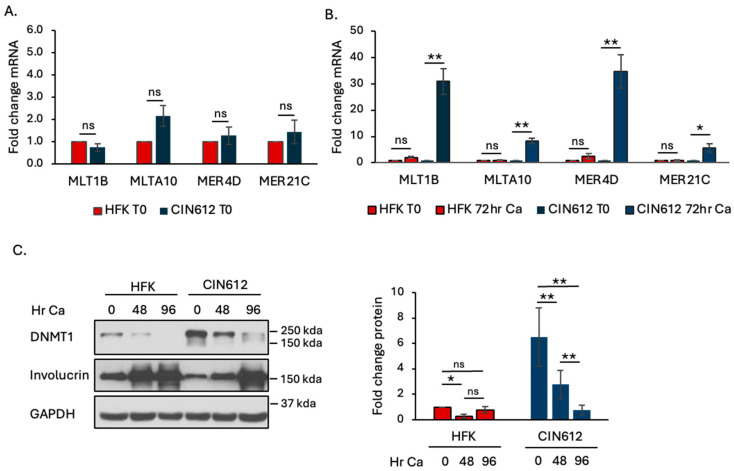
Differentiation of HPV+ cells results in an increase in ERV transcripts and a decrease in DNMT1. Protein and RNA were harvested from HFKs and CIN612 cells that were undifferentiated (T0) or differentiated in high-calcium medium for 72 h (**A**,**B**) or 48, 96 h (**C**). (**A**,**B**) qRT-PCR was performed to measure expression of MLT1B, MLTA10, MER4D, and MER21C. Fold change was calculated using the 2^−DDCT^ method. (**A**) Shown is the fold change relative to the HFK T0, set to 1. (**B**) Shown in the fold change between the HFK T0 (set to 1) and CIN612 T0 (set to 1) to their respective 72 h time points for each primer pair. Error bars represent means ± SE. Statistical significance was determined using a Student’s *t*-test. ns = not significant. * *p* ≤ 0.05; ** *p* ≤ 0.01. (**C**) Western blot analysis was carried out using antibodies to DNMT1, involucrin as a control for differentiation, and GAPDH as a loading control. Densitometry for DNMT1 was performed using ImageJ 1.54. The protein levels were normalized to GAPDH, with the HFK T0 being set to one. Shown is the average fold change of at least three independent experiments. Error bars represent means ± SE. Statistical significance was determined using a Student’s *t*-test. ns = not significant. * *p* ≤ 0.05; ** *p* ≤ 0.01. Ca = calcium.

We previously showed that differentiation of HPV+ cells results in an increase in the basal levels of IFN-β and IFN-λ which is accompanied by an increase in the basal levels of ISGs [12]. Since ERV expression can elicit an IFN response, we first wanted to determine if differentiation also triggers an increase in ERV transcription. We focused on a subset of ERVs previously shown to trigger an MDA5-MAVS-mediated IFN response: MTL1B, MLTA10, MER4D, and MER21C [30]. We examined ERV expression in uninfected human foreskin keratinocytes (HFKs) as well as HPV31+ CIN612 cells, which are derived from a CIN1 cervical lesion and maintain HPV31 episomally. We previously used CIN612 cells to show that increased caspase activity upon differentiation restrains an excessive IFN response [12]. We induced epithelial differentiation by growth in high-calcium medium, which activates the productive phase of the viral life cycle by 48 h post-exposure [38]. As shown in Figure 1A, we found the expression of this ERV subset to be similar between undifferentiated (T0) HFKs and CIN612 cells. However, upon differentiation, the level of ERV transcripts significantly increased in HPV+ cells while remaining largely unchanged in uninfected cells (Figure 1B).

Previous studies showed that MLT1B, MLTA10, MER21C, and MER4D are induced by hypomethylation through treatment with 5-aza-2-deoxcytidine [30], which mainly targets DNMT1 for degradation [39]. The HPV oncoproteins E6 and E7 have been shown to increase DNMT1 levels and activity [40,41], so we next determined if the increase in ERV expression upon differentiation correlates with changes in the levels of DNMT1. As shown in Figure 1C, we found that undifferentiated (T0) HPV31+ cells have higher levels of DNMT1 protein compared to HFKs. Upon differentiation, the levels of DNMT1 remained the same in HFKs; however, DNMT1 levels rapidly declined in HPV31+ cells. Overall, these results indicate that ERVs become transcriptionally active in HPV31+ cells upon differentiation, concomitant with a decrease in DNMT1 levels.

### 3.2. Caspase Activity Suppresses ERV Transactivation

**Figure 2:**
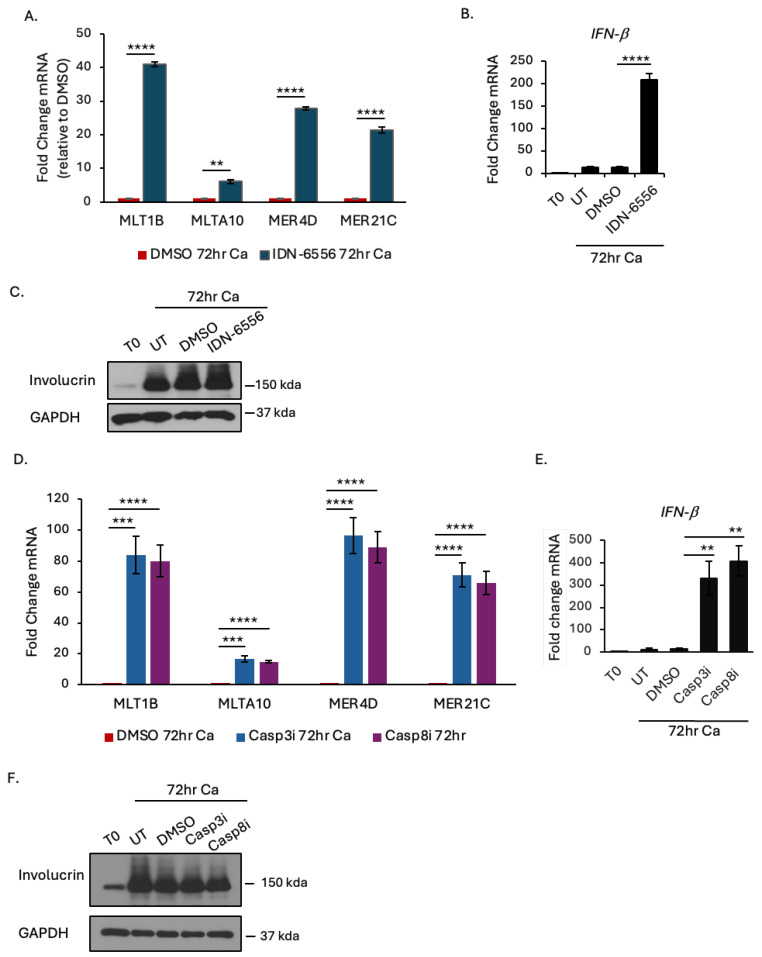
Caspase inhibition amplifies ERV expression upon differentiation. (**A**–**C**) RNA and protein were harvested from CIN612 cells that were differentiated in high-calcium medium for 72 h in the presence of DMSO or 10 μM of the pancaspase inhibitor IDN-6556. (**A**,**B**) qRT-PCR was performed to measure the expression of (**A**) MLT1B, MLTA10, MER4D, MER21C, and (**B**) IFN-b. Fold change was calculated using the 2^−DDCT^ method. Shown is the fold change relative to DMSO, set to 1 for each primer pair (**A**), or relative to T0 (**B**). Shown is the average of three independent experiments. (**C**) Western blot analysis was performed using antibodies to involucrin as a marker of differentiation and GAPDH as a loading control. Shown is a representative image of three independent experiments. (**D**–**F**) RNA and protein were harvested from CIN612 cells that were differentiated in high-calcium medium for 72 h in the presence of DMSO or 50 μM of the caspase-3 inhibitor or caspase-8 inhibitor. (**D**,**E**) qRT-PCR was carried as described for (**A**). Shown is the average of three independent experiments. (**F**) Western blot analysis was performed using antibodies to involucrin as a marker of differentiation and GAPDH as a loading control. Shown is a representative image of three independent experiments. Error bars represent means ± SE. Statistical significance was determined using a Student’s *t*-test. ns = not significant. ** *p* ≤ 0.01, *** *p* ≤ 0.001; **** *p* ≤ 0.0001 Ca = calcium.

We previously showed that treatment of differentiating HPV31+ or HPV16+ cells with the pancaspase inhibitors IDN-6556 and Z-VAD-FMK leads to an excessive increase in IFN-β and IFN-λ expression and secretion that blocks productive replication [12]. To determine if caspase inhibition further exacerbates ERV expression, we treated differentiating HPV31+ CIN612 cells with IDN-6556 for 72 h, as described previously [12]. As shown in Figure 2A,B, in addition to the expected increase in IFN-β expression, we observed a robust and significant increase in ERV transcript levels compared to the DMSO control.

We previously demonstrated that caspase-8 and caspase-3 activity is required to suppress an IFN response in differentiating HPV+ cells [12]. To determine if caspase-8 and caspase-3 also suppress ERV expression upon differentiation, we treated differentiating HPV31+ cells with specific inhibitors of caspase-8 (Z-IETD-FMK) and caspase-3 (Z-DEVD-FMK), which we previously showed induce an IFN response and block productive replication [12]. As shown in Figure 2D, inhibition of caspase-8 or caspase-3 results in a significant amplification in ERV expression compared to the DMSO control, coincident with an increase in IFN-β (Figure 2E). Importantly, and as shown previously, treatment with neither the pancaspase inhibitor nor the caspase-8 or caspase-3 inhibitors affected epithelial differentiation, as evidenced by the increase in the differentiation-dependent factor involucrin (Figure 2C,F). Overall, these results indicate that caspase-8 and caspase-3 activity restrains the transcriptional activation of at least a subset of ERVs in HPV+ cells upon differentiation.

### 3.3. ERV dsRNA Accumulates upon Caspase Inhibition

**Figure 3:**
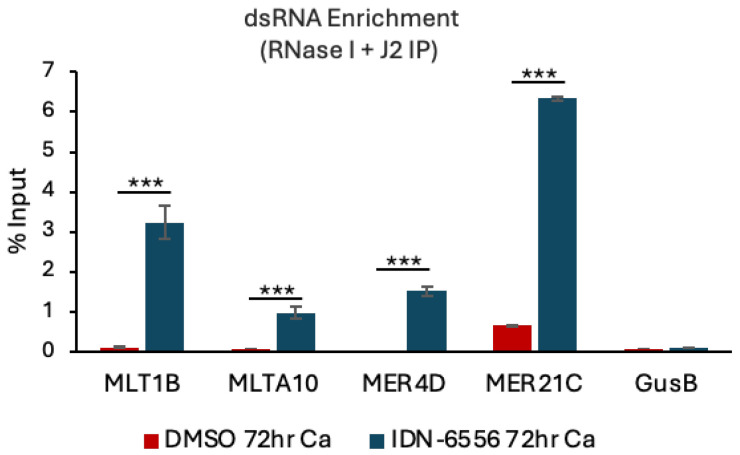
ERV dsRNA is enriched upon caspase inhibition. RNA was harvested from CIN612 cells that were differentiated in high-calcium medium for 72 h in the presence of DMSO or 10 μM of the pancaspase inhibitor IDN-6556. RNA was pre-treated with RNase I then subjected to immunoprecipitation (IP) using the dsRNA antibody J2. qRT-PCR was performed on the eluted RNA using the indicated primer pairs. dsRNA enrichment was calculated using 1% of the input RNA as a reference for each IP. The housekeeping gene GusB served as a negative control. Shown is the average of three independent experiments. Error bars represent means ± SE. Statistical significance was determined using a Student’s *t*-test. *** *p* ≤ 0.001. Ca = calcium.

Several studies have shown that ERVs exhibit bidirectional transcription and that these overlapping transcripts can pair to form dsRNAs [29,42,43,44,45]. To determine if the increase in ERV expression we observed in differentiating HPV31+ cells upon caspase inhibition results in an enrichment in dsRNA, we performed RNA immunoprecipitation (IP) coupled with quantitative PCR. We previously showed that under normal differentiating conditions, MDA5 is present at very low levels in HPV31+ cells [12], precluding us from performing IPs using an antibody to MDA5. Since attempts to overexpress MDA5 resulted in cell death, we performed IPs using the J2 antibody to specifically pull down transcripts containing dsRNA, as described previously [37,46]. Prior to IP, the RNA was treated with RNase I, which specifically degrades single-stranded RNA. As shown in Figure 3, we found that the significant increase in ERV expression in CIN612 cells upon treatment with the pancaspase inhibitor is accompanied by significant enrichment of MLT1B, MLTA10, MER21C, and MER4D by the J2 dsRNA antibody compared to the DMSO control. These results indicate that ERV transactivation and dsRNA formation is limited in differentiating HPV+ cells in a caspase-dependent manner. Furthermore, these data suggest that an increase in ERV dsRNA under caspase-deficient conditions could be a source of immunogenic RNA that triggers the MDA5-MAVS pathway.

### 3.4. ERV Expression Increases in Response to JAK-STAT Signaling

ERV expression is driven by epigenetic changes in the LTR, but also by the availability of transcription factors. Numerous ERVs have been shown to increase in expression in response to IFN and STAT1 signaling, triggering a positive feedback loop through the formation of dsRNA and engagement of RIG-I/MDA5, MAVS, TBK1, and IRF3 [37,47]. IFNs interact with their cognate receptors on the cell surface to induce JAK/STAT signaling [48]. IFN binding triggers the phosphorylation/activation of the Tyk2 and JAK1 tyrosine kinases, which in turn phosphorylate STAT1 and STAT2. Phosphorylated STAT1 and STAT2 form heterodimers that interact with IRF9 to form the ISGF3 complex, which translocates to the nucleus and binds to specific regulatory sequences, IFN-stimulated response elements (ISREs), to activate the expression of hundreds of ISGs that establish the antiviral state.

**Figure 4:**
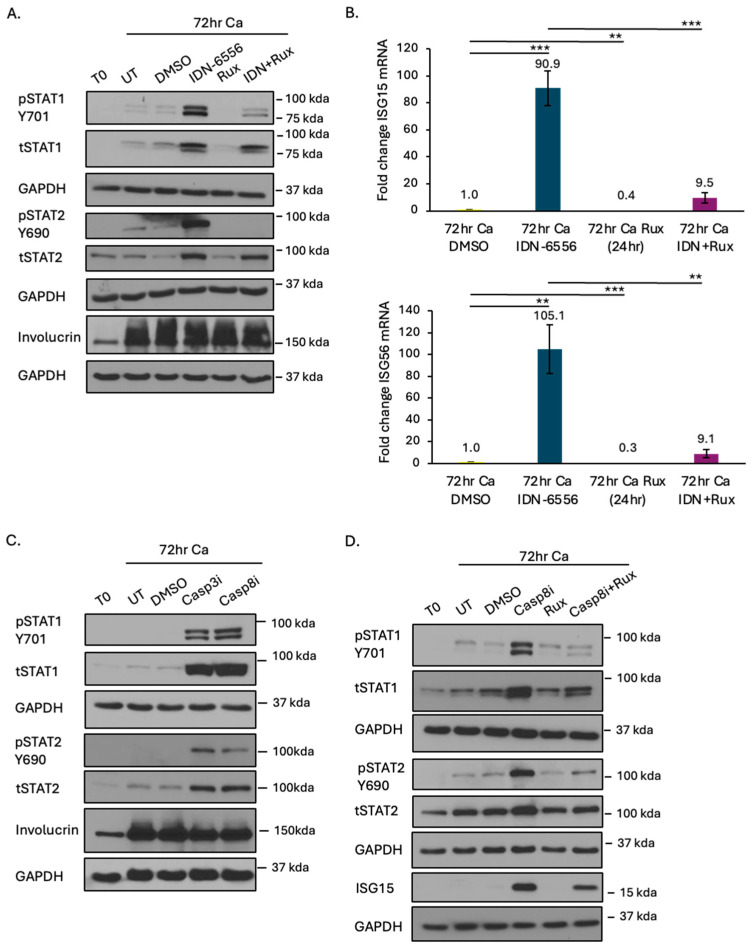
Caspase inhibition activates the JAK/STAT pathway. (**A**,**B**) Protein and RNA were harvested from CIN612 cells that were undifferentiated (T0) or differentiated in high-calcium medium for 72 h in the presence of DMSO, 10 μM of the pancaspase inhibitor IDN-6556 (IDN), 1 μM of ruxolitinib (Rux) for 24 h prior to harvest, or 10 μM IDN-6556 with 1 μM ruxolitinib added 24 h prior to harvest. (**A**) Western blot analysis was performed using the indicated antibodies, with involucrin as a marker of differentiation and GAPDH as a loading control. (**B**) qRT-PCR was performed to measure the expression of ISG15 and ISG56. Fold change was calculated using the 2^−DDCT^ method. Shown is the fold change relative to DMSO 72 h, set to 1. Shown is the average of three independent experiments. (**C**) Protein was harvested from CIN612 cells that were undifferentiated (T0) or differentiated in high-calcium medium for 72 h in the presence of DMSO, 50 μM of caspase-8 inhibitor (Z-IETD-FMK), or caspase-3 inhibitor (Z-DEVD-FMK). (**D**) Protein was harvested from CIN612 cells that were undifferentiated (T0) or differentiated in high-calcium for 72 h in the presence of DMSO, 50 μM caspase-8 inhibitor, 1 μM of ruxolitinib for 24 h prior to harvest, or 50 μM caspase-8 inhibitor with 1 μM ruxolitinib added 24 h prior to harvest. (**C**,**D**) Western blot analysis was performed using the indicated antibodies. (**A**,**C**,**D**) Shown is a representative image from three independent experiments. Error bars represent means ± SE. Statistical significance was determined using a Student’s *t*-test. ns = not significant. ** *p* ≤ 0.01, *** *p* ≤ 0.001. Ca = calcium. T = total. Rux = Ruxolitinib. IDN = IDN-6556. Casp3i = caspase-3 inhibitor. Casp8i = caspase-8 inhibitor.

We previously showed that caspase inhibition results in a significant increase in the transcript and protein levels of several ISGs (e.g., ISG56, ISG15, OAS2) in HPV+ cells upon differentiation [12]. We found the increase in ISGs occurs in an MDA5-MAVS- and IFN-dependent manner, suggesting that the JAK/STAT pathway is activated in response to caspase inhibition. To examine this directly, we measured the phosphorylation of STAT1 on Tyr701 and STAT2 on Tyr690. As shown in Figure 4A, the total and phosphorylated levels of STAT1 increased modestly upon differentiation. However, pancaspase inhibitor treatment resulted in a substantial increase in the total and phosphorylated levels of STAT1 as well as STAT2. Treatment with ruxolitinib, a JAK1/2 specific inhibitor that blocks IFN signaling, decreased phosphorylation of STAT1 and STAT2 (Figure 4A), as well as the transcript levels of ISG15 and ISG56 (Figure 4B), indicating that the IFN response induced by caspase inhibition leads to JAK/STAT pathway activation and ISG expression. Increased phosphorylation of STAT1 and STAT2 were also observed upon treatment with the caspase-8 and caspase-3 inhibitors, with ruxolitinib decreasing STAT phosphorylation and ISG15 levels cells treated with the caspase-8 inhibitor (Figure 4C,D).

**Figure 5:**
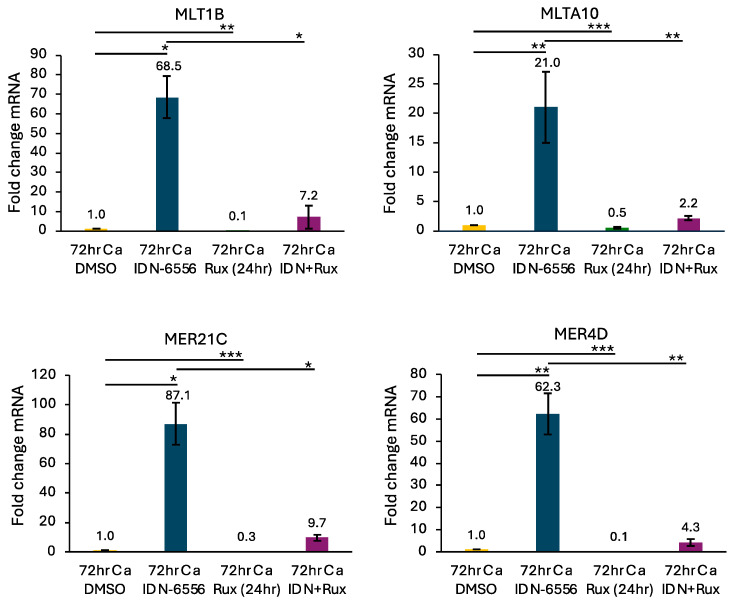
Caspase inhibition amplifies ERV expression through the JAK/STAT pathway. RNA was harvested from CIN612 cells that were differentiated in high-calcium medium for 72 h in the presence of DMSO, 10 μM of the pancaspase inhibitor IDN-6556 (IDN), 1 μM ruxolitinib (Rux) added alone 24 h prior to harvest, or IDN-6556 + 1 μM ruxolitinib added 24 h prior to harvest. qRT-PCR was performed to measure the expression of MLT1B, MLT10A, MER4D, and MER21C. Fold change was calculated using the 2^−DDCT^ method. Shown is the fold change relative to DMSO at 72 h, set to 1. Shown is the average of three independent experiments. Error bars represent means ± SE. Statistical significance was determined using a Student’s *t*-test. * *p* ≤ 0.05; ** *p* ≤ 0.01, *** *p* ≤ 0.001. Ca = calcium.

To determine if the JAK/STAT pathway contributes to the transactivation of ERVs, we treated HPV31+ cells with ruxolitinib in the presence or absence of the pancaspase inhibitor IDN-6556. As shown in Figure 5, disruption of the JAK/STAT pathway resulted in a significant decrease in the expression of MLT1B, MLTA10, MER4D, and MER21C. Overall, these results indicate that activation of the JAK/STAT pathway exacerbates ERV expression under caspase-deficient conditions, which could lead to a positive feedback loop to amplify IFN signaling.

## 4. Discussion

We previously showed that HPV induces sub-lethal apoptotic caspase activity upon differentiation to suppress a type I and type III IFN response induced by the dsRNA sensor MDA5 [12]. In this study, we have found that caspase inhibition triggers a significant increase in the expression of a subset of ERVs previously shown to induce an IFN response through dsRNA-sensing pathways. Importantly, we show that the increase in ERV expression is accompanied by an enrichment in dsRNA. Overall, these results suggest that caspase activity regulates the innate immune response in differentiating HPV+ cells via the suppression of ERVs.

ERV LTRs are typically epigenetically silenced to prevent unregulated expression that can induce genomic instability and tumorigenesis [49]. Our data indicate that ERV expression increases in HPV+ cells upon differentiation. DNA methylation at CpGs represents a major mechanism of transcriptional control of ERVs [50]. The subset of ERVs we examined has been shown to increase in response to hypomethylation induced by depletion of DNMT1 or treatment with 5-azacytidine [29,30]. Our observation that DNMT1 levels are higher in undifferentiated HPV+ cells compared to HPV- cells is consistent with previous studies showing that the HPV oncoproteins E6 and E7 increase DNMT1 levels and activity [31]. However, we have found that DNMT1 levels decrease in HPV+ cells upon differentiation, raising the possibility that ERV LTRs become demethylated, leading to increased transcription and dsRNA formation that initiates an IFN response. However, it is possible that other epigenetic mechanisms to repress ERVs, such as histone modifications, are lost upon differentiation as well as in response to caspase inhibition. Further studies are needed to understand if different epigenetic marks work in tandem to silence ERV elements and whether epigenetic dysregulation upon differentiation contributes to ERV expression.

ERV RNA, DNA, and protein products can serve as viral agonists of PRR-signaling pathways, inducing an innate immune response in a manner similar to exogenous viruses [25]. The antiviral state induced by ERV products is referred to as viral mimicry [51]. Our data indicate that caspase-8 and caspase-3 play a critical role in suppressing aberrant ERV transcription upon differentiation of HPV+ cells. Our finding that the increase in ERV expression upon pancaspase inhibition is accompanied by an accumulation of ERV dsRNA supports the idea that ERV RNA serves as a viral mimicry “driver” of the IFN response through the MDA5/MAVS pathway. Although the subset of ERVs we examined has been shown to induce an antiviral response through formation of dsRNA [30], we cannot rule out the possibility that other retroelements contribute to the induction of the IFN response elicited by caspase inhibition. In addition to ERV LTR elements, the non-LTR transposable elements long-interspersed (LINEs) and short-interspersed elements (SINEs) (e.g., Alu elements) may also be sources of cytosolic dsRNA that stimulate and IFN response through viral mimicry [52,53,54]. A more comprehensive examination of how retroelement expression changes in response to caspase inhibition and how these changes impact the antiviral response in HPV+ cells will be the focus of future studies.

Our data indicate that activation of the JAK/STAT-signaling pathway is necessary for the amplification in ERV expression induced by caspase inhibition. Previous studies showed that HPV31 blocks activation of the JAK/STAT pathway in undifferentiated cells by transcriptionally silencing STAT1, in part through DNA methylation [55]. Although total STAT1 and phosphorylated STAT1 levels increase moderately upon differentiation, these results indicate the transcriptional repression of STAT1 can be overcome. Whether the increase in total STAT1 is due to epigenetic changes associated with a decrease in DNMT1 is currently unclear. The JAK/STAT pathway is well established to mediate the IFN response through the expression of ISGs [48]. ISGs are regulated by *cis*-regulatory elements that are bound by IRF and STAT transcription factors upon activation of IFN-signaling pathways. Many ERVs contain binding sites for IFN-inducible transcription factors, such as IRFs and STATs, in their LTRs [43,47,56]. Our finding that MTL1B, MLTA10, MER4D, and MER21C increase in a JAK/STAT-signaling-dependent manner indicate that they are IFN-inducible. We previously showed that caspase inhibition results in a substantial increase in MDA5 [12]. The increased levels of MDA5 may create a cellular state primed to respond to the accumulation of ERV dsRNA, resulting in an amplification loop of IFN signaling.

In addition to activating an antiviral response, numerous ERV groups have been shown to play a functional role in regulating innate immune pathways. Most ERVs exist as solo LTRs due to homologous recombination between the 5′ and 3′ LTRs [57,58]. ERV LTRs contain binding sites for a wide variety of transcription factors, and several studies have provided evidence that ERV LTRs influence the expression of nearby genes involved in immune responses, especially those related to IFN signaling [25,43,47,56,59]. Furthermore, ERV-derived long non-coding RNAs (lncRNA) can act as positive regulators of the IFN response [60]. The increase in ERV expression induced by caspase inhibition therefore has the potential to trigger specific gene networks in HPV+ cells. In support of this, a recent study showed that differentially expressed ERVs in cervical cancers correlate with changes in nearby genes involved in immunity [61]. A more complete understanding of how ERV reactivation influences the innate immune response in HPV+ cells will be a focus of future studies.

Our studies suggest that caspase activity facilitates productive replication by suppressing the accumulation of ERV dsRNAs that can drive an MDA5-dependent IFN response. However, infection with many exogenous viruses has been shown to result in ERV reactivation, which in turn co-contributes to viral diseases as well as viral malignancies [28,62]. KSHV has been shown to transactivate ERV-K, with the ERV-K accessory protein K9 being involved in KSHV pathogenesis and tumorigenesis [63]. EBV has been shown to transcriptionally activate the ERV-K18 *env* gene, which exhibits superantigen activity and may play a role in facilitating EBV transmission as well as in enhancing the EBV-specific T cell response [64]. Expression of ERVs has been detected in numerous human cancers, including cervical cancer [61,65,66]. However, little is known regarding how ERV expression is impacted by HPV during the viral life cycle. Whether changes in ERV expression patterns upon differentiation of HPV+ cells, with and without caspase inhibition, could indicate potential functions of these elements in the viral life cycle will be an interesting area of future research.

## Data Availability

The original contributions presented in this study are included in the article. Further inquiries can be directed to the corresponding author.
